# Tissue interactions in the developing chick diencephalon

**DOI:** 10.1186/1749-8104-2-25

**Published:** 2007-11-13

**Authors:** Maria Flavia Guinazu, David Chambers, Andrew Lumsden, Clemens Kiecker

**Affiliations:** 1 MRC Centre for Developmental Neurobiology, Guy's Hospital Campus, King's College, London SE1 1UL, UK

## Abstract

**Background:**

The developing vertebrate brain is patterned first by global signalling gradients that define crude anteroposterior and dorsoventral coordinates, and subsequently by local signalling centres (organisers) that refine cell fate assignment within pre-patterned regions. The interface between the prethalamus and the thalamus, the *zona limitans intrathalamica *(ZLI), is one such local signalling centre that is essential for the establishment of these major diencephalic subdivisions by secreting the signalling factor Sonic hedgehog. Various models for ZLI formation have been proposed, but a thorough understanding of how this important local organiser is established is lacking.

**Results:**

Here, we describe tissue explant experiments in chick embryos aimed at characterising the roles of different forebrain areas in ZLI formation. We found that: the ZLI becomes specified unexpectedly early; flanking regions are required for its characteristic morphogenesis; ZLI induction can occur independently from ventral tissues; interaction between any prechordal and epichordal neuroepithelial tissue anterior to the midbrain-hindbrain boundary is able to generate a ZLI; and signals from the dorsal diencephalon antagonise ZLI formation. We further show that a localised source of retinoic acid in the dorsal diencephalon is a likely candidate to mediate this inhibitory signal.

**Conclusion:**

Our results are consistent with a model where planar, rather than vertical, signals position the ZLI at early stages of neural development and they implicate retinoic acid as a novel molecular cue that determines its dorsoventral extent.

## Background

Global signalling gradients regionalise the emerging vertebrate brain at the earliest stages of neural development [1-3]. This crude initial pattern is subsequently refined by the activity of local signalling centres [4-6]. Dorsoventral (DV) neural patterning is regulated by two such signalling centres, the roof plate and the floor plate, which stretch along the dorsal and ventral midlines of the neural tube, respectively [7-9]. Anteroposterior (AP) regionalisation is regulated by several discrete signalling centres, such as the anterior neural border [10-13], the midbrain-hindbrain boundary (MHB) [14-16], rhombomere 4 in the hindbrain [17,18] and, subsequently, the boundaries between rhombomeres [19,20]. Recently, we and others have shown that the interface between the prethalamus (Pth) and the thalamus (Th), the *zona limitans intrathalamica *(ZLI), also acts as a signalling centre that is essential for the establishment of these major diencephalic subdivisions [21-23].

For a considerable time during development, the ZLI is the only structure in the alar part of the neural tube that expresses the secreted signalling factor Sonic hedgehog (Shh) [24]. Elsewhere at early stages, *Shh *is expressed along the ventral midline of the neural tube, and one of its best-characterised functions is the dose-dependent induction of ventral cell fates in hindbrain and spinal cord [25]. In the diencephalon, Shh is required for both proliferation and the establishment of regionally specific gene expression, and the ZLI provides a major source of this signal that induces a differentiation switch in cells of the flanking regions, the Pth anteriorly and the Th posteriorly [21-23,26-28]. The differential response of Pth and Th to ZLI signalling is regulated by a prepattern of transcription factors – most notably Irx3, which is expressed posterior to the ZLI and directs Th identity upon receipt of the Shh signal [21].

The first clues as to what mechanisms underlie ZLI induction and formation have already been revealed. Thus, it has been suggested that the site of prospective ZLI formation is marked by the interface of the expression domains of *Six3 *and *Irx3 *[29]. Both genes are regulated by the Wnt/β-catenin signalling pathway during gastrulation [30,31]; hence, the ZLI may be positioned by a specific threshold of the Wnt activity gradient that polarises the AP axis of the early neural plate [2,3,32], similar to what has been proposed for the MHB [33,34]. This idea is supported by the profound disorganisation of the diencephalon in mice lacking the Wnt co-receptor LRP6 [35]. A recent fate mapping study in zebrafish also indicates that a considerable diencephalic pre-pattern is already set up during gastrulation [36]. Furthermore, the ZLI is derived from a wedge-shaped area in the early prosencephalon that is confined by cell lineage-restriction boundaries and is characterised by a gap in the expression of *Lunatic fringe *(*Lfng*) [37], but how the formation of this wedge-shaped presumptive ZLI relates to the expression of *Six3 *and *Irx3 *remains unclear. The zinc finger transcription factors Fez and Fez-like (Fezl) are both expressed in the prospective Pth, and *Fez*/*Fezl *double mutant mice lack both Pth and ZLI [38]. The Th is also reduced in such mice, probably attendant on the lack of a ZLI and local Shh signalling. Ectopic expression of *Fezl *within the Th primordium results in a posterior misplacement of the ZLI and in a repression of *Irx1*, the putative functional orthologue of *Irx3 *in the mouse. Similarly, *fezl *is essential for Pth formation in zebrafish embryos [39]. These data suggest that Fez and Fezl determine the anterior limit of ZLI formation. Recent work in zebrafish indicates that *otx1/2 *expression is required for ZLI formation while *irx1b *expression suppresses it [40]. Thus, the ZLI may form in an *otx*-positive corridor that is bound anteriorly by *Fez*/*Fezl *expression and posteriorly by *Irx *expression.

Although the ZLI merges with the floor plate ventrally, and although both structures share a characteristic set of marker genes, cell labelling experiments have excluded the possibility that the ZLI forms as an extension of the floor plate through dorsal migration or expansion of ventral cells [23,41]. These experiments strongly suggest a requirement for inductive signals in ZLI formation. While it has been proposed that a ventral-to-dorsal relay of Shh signalling in the diencephalon underlies ZLI formation [41], observations in zebrafish embryos are difficult to reconcile with a requirement for ventral neural tissues during this process [23]. Furthermore, an inhibitory influence of dorsal diencephalic tissue on ZLI formation has been described [41].

The observations described above have started to address the question of ZLI initiation; but we still lack a general picture of how this important signalling centre is established. Here, we have performed tissue culture experiments with chick forebrain explants to characterise the roles of various early brain regions during ZLI formation. Using diencephalic explants, we found that the ZLI becomes specified unexpectedly early and that flanking regions are required for its characteristic morphogenesis. Co-culture of neural explants revealed that ZLI induction can occur independently from ventral tissues and that any interaction between prechordal tissue and epichordal tissue anterior to the MHB generates a ZLI, suggesting that the entire forebrain-midbrain area is competent to form a ZLI and that planar signals are likely to be involved in ZLI positioning. Furthermore, we have confirmed that signalling from the dorsal diencephalon antagonises ZLI formation [41] and provide evidence that localised production of retinoic acid (RA) in the epithalamus may mediate this inhibition. Our results provide novel insights into the timing, localisation and molecular nature of ZLI formation.

## Results

During embryonic development, cells and tissues from a single origin gradually acquire different identities. Two conceptually important steps in this narrowing of developmental potential are the specification and the determination of tissue fate. A tissue has become specified once it is able to differentiate according to its fate when it is explanted from the embryo and kept in a neutral environment, such as serum-free tissue culture. A tissue has become determined if it will differentiate according to its fate even after it has been grafted to an ectopic location in the embryo where other patterning influences may pertain. To date, it remains largely unknown when the ZLI is induced and what mechanisms underlie its specification.

The explantation of tissues and their culture *in vitro *is a classic approach to study their specification, inductive interactions between them and the influence of soluble factors on their differentiation (for example, [42]). Therefore, we established a method for the culture of chick forebrain explants in order to determine when the ZLI becomes specified. We tested various culture media and found that Neurobasal medium allowed us to keep neural tissue in culture under serum-free conditions for up to four days. Examination of representative explants by electron microscopy revealed the ultrastructural characteristics of proliferating tissue, such as the presence of actively dividing cells, de-condensed chromatin in the majority of cell nuclei and an abundance of cellular organelles in the supranuclear cytoplasm of many cells (not shown), indicating that our culture conditions were suitable to maintain the viability of explants for the time required to study inductive interactions in the forebrain.

### The ZLI is specified before stage HH10

**Figure 1:**
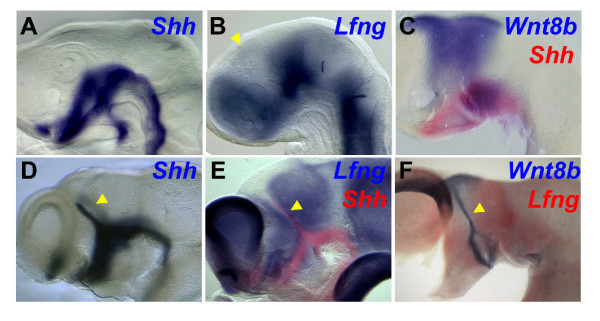
Gene expression in the diencephalon during ZLI formation. **(A-F) ***In situ *hybridisation was used to analyse the expression of *Shh *(blue in (A, D); red in (C, E)), *Lfng *(blue in (B, E; red in (F)) and *Wnt8b *to stage HH14 (A, B), HH15 (C), HH18.5 (D) and HH22 (E, F) chick embryos. Whole heads are shown in (A-D) and hemisected brains in (E, F) (anterior to the left, dorsal to the top). Note expression of *Shh *and *Wnt8b *in the ZLI (yellow arrowheads in (B, D-F)).

**Figure 2:**
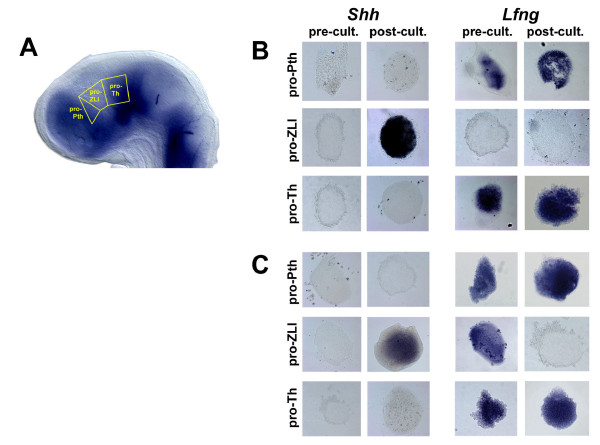
The ZLI is specified before stage HH10. **(A) **Explanted brain regions are schematically shown in a stage HH14 chick brain stained by *in situ *hybridisation for the expression of *Lfng *(anterior to the left, dorsal to the top). **(B) **Pro-Pth, pro-ZLI and pro-Th explants from stage HH14 brains were analysed by *in situ *hybridisation for the expression of *Shh *(left) and *Lfng *(right) at the time of dissection (pre-cult.) or after culture for 24 hours (post-cult.). Note induction of *Shh *in cultured pro-ZLI explants and absence of *Lfng *expression in pro-ZLI explants at the time of dissection and after culture. **(C) **Pro-Pth, pro-ZLI and pro-Th explants from stage HH10 brains. Note induction of *Shh *and absence of *Lfng *expression in cultured pro-ZLI explants. *Lfng *is expressed in pro-ZLI explants at the time of dissection.

In chick, the definitive ZLI expresses *Shh *along its full DV extent from embryonic stage HH18 onwards; however, the area that gives rise to the ZLI is characterised by the absence of *Lfng *expression from HH12 onwards (Figure 1) [37]. Hence, the first steps of ZLI induction must occur before this stage. In order to define when ZLI tissue becomes specified, we dissected a lateral stripe of neural tissue from the anterior neural tube of HH14 embryos and excised pieces at the level of the prospective Pth (pro-Pth), of the *Lfng*-free wedge (from here on referred to as pro-ZLI) and of the prospective Th (pro-Th) as indicated in Figure 2a. We specifically avoided including tissue from the basal plate and the dorsal midline of the neural tube. Explants from one side of the neural tube were fixed immediately while the corresponding explants from the opposite side were cultured for 24 hours, after which un-manipulated control embryos had developed to stage HH19-20 and showed the characteristic peak of *Shh *expression that marks the definitive ZLI (Figure 1 and data not shown). Both groups of explants were analysed for the expression of *Lfng *and *Shh*. As expected, *Lfng *was expressed in both pro-Pth (16/20) and pro-Th (18/20) explants but was absent from pro-ZLI explants at the time of dissection and after culture (20/20; Figure 2b). This observation is consistent with the gap of *Lfng *expression that characterises the ZLI *in vivo *from HH13 onwards and confirms the accuracy of our dissections. At the time of dissection, no explant expressed *Shh*, consistent with the absence of *Shh *expression from alar neural tissue before ZLI formation *in vivo*. After culture, neither Pth (18/20) nor Th (18/20) explants expressed *Shh *but ZLI explants showed strong *Shh *expression (20/20; Figure 2b), indicating that our explants had followed the normal temporal dynamics of *Shh *expression *in vivo *and that the ZLI is specified before HH14.

Equivalent dissections were performed with HH10 embryos and yielded comparable results – the only difference was that pro-ZLI explants expressed *Lfng *at HH10, as the *Lfng*-negative wedge has not yet formed at this stage (*Lfng*: 16/20 pro-Pth, 20/20 absent from pro-ZLI, 16/20 pro-Th; *Shh*: 18/20 absent from pro-Pth, 20/20 pro-ZLI, 17/20 absent from pro-Th; Figure 2c). Pro-ZLI explants also expressed *Wnt8b *(a marker of the *Lfng*-free area [5]) at the time of dissection and after culture, but they did not express *Dlx2 *(Pth marker) or *Gbx2 *(Th marker) after 72 hours of culture, indicating that they were free from pro-Pth and pro-Th tissue (data not shown). The small size of the diencephalic primordium in embryos younger than HH10 imposes a practical limit to our experimental approach and prevents the reliable dissection of pro-Pth, pro-ZLI and pro-Th explants without contamination by surrounding tissue. In summary, our results indicate that the ZLI (as marked by *Shh *expression) is specified before HH10 – long before the onset of *Shh *expression in this signalling centre and even before the downregulation of *Lfng *in the pro-ZLI.

### The ZLI forms independently from the basal neural tube after stage HH10

**Figure 3:**
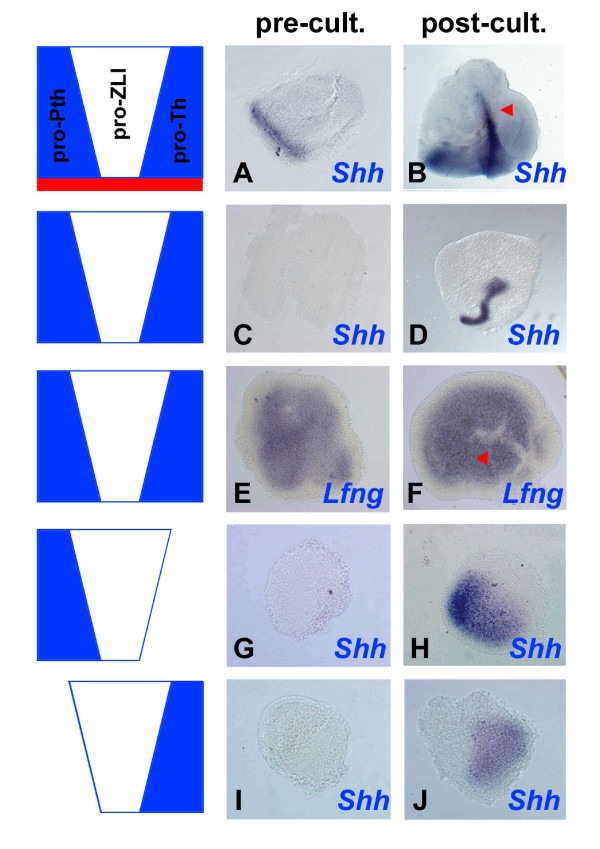
ZLI formation occurs independently from ventral tissues after stage HH10 and ZLI morphogenesis depends on the integrity of its flanking regions. Diencephalic explants were taken from **(A-F) **stage HH10 and **(G-J) **stage HH14 embryos and were analysed by *in situ *hybridisation for the expression of *Shh *(a-d, g-j) and *Lfng *(E, F) at the time of dissection (pre-cult.) or after culture for 48 (B, D, J) or 24 hours (F) (post-cult.). (A, B) Explants comprising pro-Pth, pro-ZLI, pro-Th and basal tissue (red). Note basal expression of *Shh *at the time of dissection and formation of a ZLI after culture (red arrowhead). (C, D) Pro-Pth + pro-ZLI + pro-Th explants excluding basal tissue. Note absence of *Shh *expression at the time of dissection and induction of a ZLI after culture. (E, F) Explants as in (C, D). Note formation of a *Lfng*-free stripe of cells after culture (red arrowhead). (G, H) Pro-Pth + pro-ZLI explants; (I, J) pro-ZLI + pro-Th explants. Note induction of *Shh *in a patch, rather than a line, after culture (compare to (B, D)).

The robust induction of *Shh *in pro-ZLI explants that are free from basal neural tissue suggests that ZLI formation does not depend on ventral signals after HH10. We confirmed that ZLI formation can occur independently from ventral tissues in larger explants that encompassed the entire thalamic anlage (pro-Pth + pro-ZLI + pro-Th). Such explants expressed *Shh *in a narrow stripe after 24 hours in culture and this expression was mirrored by a gap in *Lfng *expression (*Shh*, 7/8; *Lfng*-free stripe, 3/3; Figure 3a–f). Importantly, ZLI formation occurred both in explants that included the basal plate (4/4) and in explants from which basal tissue had been removed (3/4; compare Figure 3a, b with Figure 3c, d). Thus, the alar plate is sufficient to maintain a program of ZLI formation after stage HH10.

### Flanking tissues are required for ZLI morphogenesis

Pro-ZLI explants recapitulate the temporal progression of *Shh *expression of the corresponding area *in vivo *(Figure 2b, c). However, ZLI formation is also characterised by the striking metamorphosis of the wedge-shaped *Lfng*-negative area into the narrow band of cells that expresses *Shh *at later stages. While this aspect of ZLI formation does not become evident from the culture of pro-ZLI explants, it is faithfully mimicked by the larger Pth/ZLI/Th explants that typically form a narrow line of *Shh *expression (7/8; Figure 3a–d). This observation suggests that flanking tissues are involved in regulating ZLI morphogenesis. Furthermore, *Shh *is expressed in a patch rather than a narrow line in explants containing the pro-ZLI region and either the pro-Pth only (pro-Pth + pro-ZLI; 4/5; Figure 3g, h) or the pro-Th only (pro-ZLI + pro-Th; 4/5; Figure 3i, j). This suggests that the integrity of the entire Pth/ZLI/Th region is required for proper ZLI morphogenesis.

### Interaction of prechordal and epichordal neural tissue results in ZLI induction

**Figure 4:**
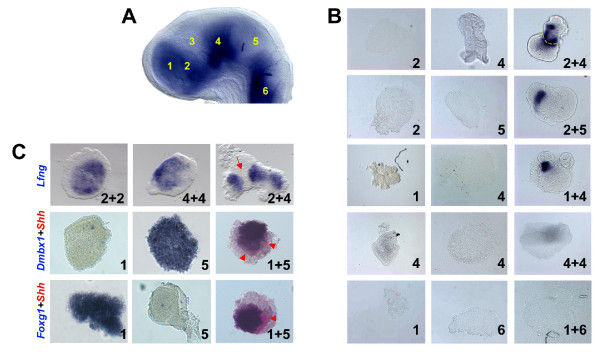
Co-culture of prechordal and epichordal neural explants results in ZLI induction. **(A) **Explanted brain regions are schematically shown in a stage HH14 chick brain stained by *in situ *hybridisation for the expression of *Lfng *(anterior to the left, dorsal to the top): 1, telencephalon; 2, pro-Pth; 3, pro-ZLI; 4, pro-Th; 5, midbrain; 6, hindbrain. **(B) **Explants from brain regions as indicated in (A) were cultured in isolation (left two columns) or in combination (right column) for 24 hours and analysed for the expression of *Shh *by *in situ *hybridisation. Note induction of *Shh *at the interface of co-cultured pro-Pth + pro-Th, pro-Pth + midbrain and telencephalon + pro-Th tissue, but not after co-culture of pro-Th + pro-Th or telencephalon + hindbrain. The yellow broken line in panel '2 + 4' indicates the interface between pro-Pth and pro-Th tissue. **(C) **Explants from brain regions as indicated in (A) were cultured in isolation (left two columns) or in combination (right column) for 24 hours and analysed for the expression of *Lfng, Dmbx1, Foxg1 *(all in blue) and *Shh *(red) by *in situ *hybridisation. Note gap of *Lfng *expression in '2 + 4' explants (red arrow) and induction of *Shh *in '1 + 5' explants (red arrowheads).

It has been suggested that the ZLI, similar to the MHB [33,34], is positioned anteroposteriorly as a direct readout of a specific threshold in the Wnt signalling gradient that regulates AP patterning during gastrulation [5,30] and that it marks the interface between the prechordal and the epichordal central nervous system [43]. The border between the expression domains of *Six3 *(anteriorly) and *Irx3 *(posteriorly) seems to presage the site of ZLI formation [29]. These observations suggest that planar signals are crucial for positioning the ZLI, prompting us to ask whether the apposition of anterior (prechordal) and posterior (epichordal) neural tissue results in ZLI induction. Prechordal explants (prospective telencephalon, pro-Pth) and epichordal explants (pro-Th, prospective midbrain) were dissected from stage HH14 embryos (Figure 4a), labelled with red and green CellTracker dyes, respectively (to be able to distinguish their origin after culture), and were cultured in combination. Any combination of prechordal and epichordal neuroepithelium resulted in induction of *Shh *expression at the junction of the two explants (pro-Pth + pro-Th, 7/7; pro-Pth + midbrain, 6/6; telencephalon + pro-Th, 7/8; Figure 4b). In the majority of these co-cultures, *Shh *expression was observed either in the prechordal or in the epichordal explant while a few expressed *Shh *in both (Figure 4b, panel '2 + 4'), indicating that both prechordal and epichordal tissue are susceptible to ZLI induction. However, *Shh *expression was not detected in prechordal/prechordal or epichordal/epichordal (11/12) co-cultures. Furthermore, co-cultures of prechordal and hindbrain explants did not result in induction of *Shh *expression (11/11; Figure 4b). Taken together, these experiments suggest that a planar interaction between prechordal and epichordal tissue is sufficient to induce ZLI identity. Both prechordal and rostral epichordal (posterior diencephalon, midbrain) tissue are competent to form a ZLI, but more caudal neuroepithelium is not competent to mediate this induction.

Apart from *Shh *induction, ZLI formation is hallmarked by the downregulation of *Lfng *[37]. Co-culture of prechordal and epichordal tissue results in aggregates with a characteristic gap in *Lfng *expression at the junction between the two tissues (8/12; Figure 4c) or downregulate *Lfng *expression completely in the smaller explant (4/12) while aggregates that consist of only one type of tissue do not display a stripe of *Lfng *downregulation (0/16). This observation lends further support to the idea that prechordal/epichordal interactions result in ZLI formation.

The induction of a ZLI-like structure at the interface between telencephalic and mesencephalic explants raises the question to what extent these tissues maintain their respective regional identities. Midbrain explants express *Dmbx1 *(14/16) while telencephalic explants express *Foxg1 *(13/16). The expression of both genes is maintained in the corresponding tissues in telencephalon + midbrain co-cultures that also show *Shh *induction (Foxg1, 11/16; Dmbx1, 12/16; Figure 4c), suggesting that they maintain their regional identities and that no re-specification has occurred at the time of *Shh *induction.

**Figure 5:**
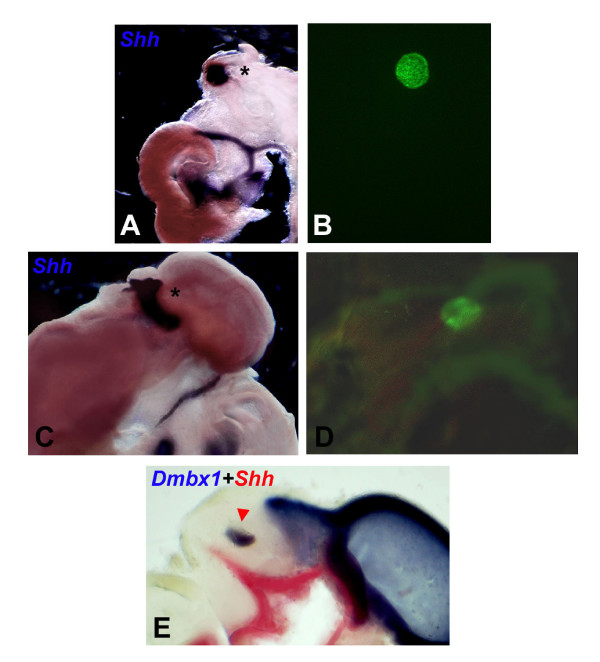
Prechordal-epichordal interactions anterior to the MHB result in ZLI induction *in vivo*. **(A) **Ectopic induction of *Shh *following transplantation of quail pro-Pth tissue into chick midbrain at stage HH10. **(B) **QCPN antibody staining allowed for localisation of the graft (asterisk in (A)). **(C) **Ectopic induction of *Shh *following transplantation of quail telencephalic tissue into chick midbrain at stage HH10. **(D) **QCPN antibody staining allowed for localisation of the graft (asterisk in (C)). **(E) **A graft of quail midbrain tissue into a chick pro-Th at stage HH10 maintains midbrain identity (red arrowhead; *Dmbx1 *expression in blue), but does not result in ectopic induction of *Shh *(red).

To test whether the interaction between prechordal and epichordal neuroepithelium is sufficient for ZLI induction we grafted neural explants from quail embryos to different locations in the neural tube of chick embryos at stage HH10. Chimaeric embryos were cultured for four days, fixed, and subjected to *in situ *hybridisation for *Shh *expression and to immunohistochemical staining with a quail-specific antibody (QCPN) to allow localisation of the graft. Embryos that showed poor integration of the grafted tissue or structural anomalies were excluded from subsequent analysis. Grafts of telencephalic or Pth tissue into the prospective Th or midbrain resulted in induction of *Shh *expression (10/12) while such grafts failed to induce *Shh *expression when transplanted into the telencephalon (1/6, likely a dissection artifact), the Pth (0/6) or the hindbrain (0/6; Figure 5a–d). Conversely, Th or midbrain tissue elicited *Shh *expression when grafted into the telencephalon or the Pth (6/6; however, these inductions were accompanied by severe malformations and structural abnormalities), but no *Shh *expression was detected when they were grafted into the Th (0/9), the midbrain (0/9) or the hindbrain (0/6; Figure 5e). These results confirm that the apposition of any prechordal and epichordal tissue anterior to the hindbrain results in ZLI formation and are in line with another recently published paper [22].

### The ZLI regulates thalamic gene expression in explant cultures

**Figure 6:**
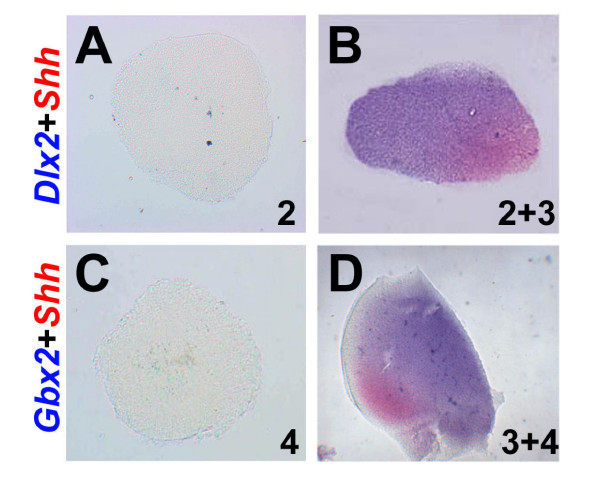
The ZLI is necessary and sufficient for the expression of thalamic differentiation genes in explant culture. **(A) **Pro-Pth and **(B) **pro-ZLI + pro-Pth explants were isolated from stage HH10 embryos, cultured for 72 hours and analysed for the expression of *Shh *(red) and *Dlx2 *(blue). **(C) **Pro-Th and **(D) **pro-ZLI + pro-Th explants were isolated from stage HH10 embryos, cultured for 72 hours and analysed for the expression of *Shh *(red) and *Gbx2 *(blue).

Shh signalling from the ZLI has been demonstrated to regulate regional identity in Pth and Th in various experimental systems [21-23,28]. To confirm whether this function is maintained in our culture system, we isolated pro-Pth and pro-Pth + pro-ZLI explants, cultured them for 72 hours (to approximately stage HH24) and analysed them by double *in situ *hybridisation for the expression of *Shh *and the Pth marker *Dlx2*. Pro-Pth explants do not express *Shh *(0/30) and *Dlx2 *only weakly in a few cases (7/30) after 72 hours in culture (Figure 6a) while pro-ZLI + pro-Pth explants express both genes in adjacent domains under these conditions (23/30; Figure 6b). In the complementary experiment, pro-Th explants do not express *Shh *(0/30) and only rarely the Th marker *Gbx2 *(3/30) after 72 hours in culture (Figure 6c) while pro-ZLI + pro-Th explants express both genes in the majority of cases (25/30; Figure 6d). This experiment confirms that our ZLI explants maintain their function as a thalamic 'organiser' and demonstrate that the ZLI is both necessary and sufficient to induce regional identity in Pth and Th under these conditions.

### Dorsal diencephalic tissue antagonises ZLI formation

**Figure 7:**
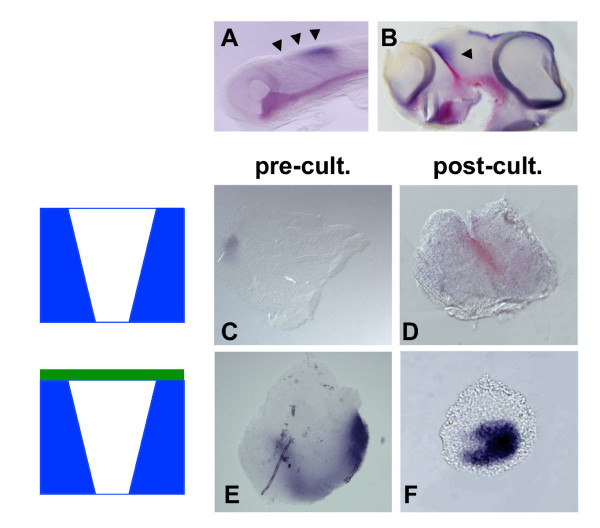
Dorsal diencephalic tissue suppresses ZLI formation. **(A, B) **Whole embryos and **(C-F) **explants were analysed by double *in situ *hybridisation for expression of *Shh *(red) and *Wnt3a *(blue). (A) Embryo at stage HH10, corresponding to the time when explants were dissected. Note expression of *Wnt3a *along the dorsal midline of the diencephalon and midbrain (black arrowheads). (B) Embryo at stage HH23, correponding to the developmental stage of explants after culture for 72 hours. Note expression of *Wnt3a *in a dorsal triangluar domain flanking the ZLI posteriorly (black arrowhead). (C) Explants excluding dorsal diencephalic tissue express only little *Wnt3a *at the time of explantation (pre-cult.) and form a ZLI after 72 hours in culture (D) (post-cult.). (E) Explants including dorsal diencephalic tissue express high levels of *Wnt3a *at the time of dissection and after culture but fail to express *Shh *after culture (F).

Planar interactions between prechordal and epichordal neural tissue result in ZLI formation while ventral signals are dispensable for ZLI formation after stage HH10 – although they may be required at earlier stages [41]. To analyse a potential effect of dorsal neural tissue on ZLI formation, we cultured Pth/ZLI/Th explants including the dorsal part of the diencephalon, the epithalamus, using *Wnt3a *as a marker of dorsal diencephalic identity (Figure 7). After culture, *Shh *expression was absent (3/5) or significantly reduced (2/5) in such explants compared with explants from which the prospective epithalamus had been removed (4/4; compare Figure 7f with 7d). This finding, consistent with the results in another recent study [41], suggests that dorsal diencephalic tissue counteracts ZLI formation.

### Retinoic acid is a dorsal ZLI inhibitor

**Figure 8:**
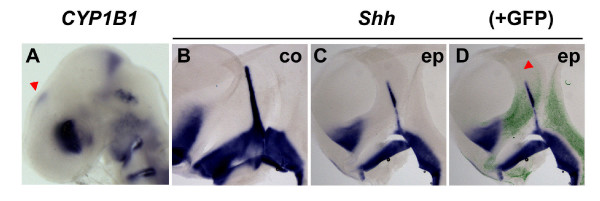
*CYP1B1 *is expressed in the dorsal diencephalon and is able to suppress ZLI formation. **(A) **Stage HH15 chick head, *in situ *hybridisation for expression of *CYP1B1*. Note expression in the dorsoanterior part of the eye, anterior to the MHB and in the epithalamus (red arrowhead). **(B-D) **Stage HH16 embryos were electroporated with CYP1B1-IRES-GFP, cultured to stage HH21/22 and analysed by *in situ *hybridisation for expression of *Shh *followed by immunochemical detection of green flourescent protein (GFP; lateral views of hemisected brains, anterior to the left, dorsal to the top). (B) Non-electroporated control side (co). (C, D) Electroporated side (ep); (D) an overlay with the anti-GFP fluorescent signal in green. Note reduced *Shh *expression in the ZLI even in areas where only few cells are transfected (red arrowhead), suggesting a non-autonomous effect.

The observation that dorsal diencephalic tissue inhibits ZLI formation raises the question as to which molecular signal exerts this negative influence. We have recently identified the cytochrome P450 enzyme CYP1B1 in a microarray screen for genes that are differentially expressed in hindbrain rhombomeres (Chambers *et al*., in preparation). In chick, *CYP1B1 *transcripts are enriched at the MHB and biochemical analyses as well as embryological data have indicated that CYP1B1 efficiently catalyses RA production *in vitro *and *in vivo *[44], unlike members of the CYP26 family that function by degrading RA [45]. The observation that *CYP1B1 *is also expressed in the epithalamus from stage HH15 onwards (Figure 8a) prompted us to ask whether RA might be the dorsal signal that inhibits *Shh *expression in the ZLI. Overexpression by *in ovo *electroporation of a CYP1B1 expression plasmid into the diencephalon of stage HH16 embryos leads to a dramatic reduction of *Shh *expression on the electroporated side (14/18; Figure 8b, c). Downregulation of *Shh *not only occurred in electroporated cells themselves, but also in neighbouring tissue, indicating a cell non-autonomous effect and suggesting the involvement of a diffusible signal such as RA (Figure 8d). Our results indicate that RA, generated by the activity of CYP1B1 in the epithalamus from stage HH15 onwards, is a candidate signal to mediate the dorsal repression of ZLI formation.

## Discussion

The central nervous system is progressively regionalised by successive and simultaneous extracellular signals, resulting in a gradual diversification of cellular fates [46]. At early stages of development, during gastrulation, the emerging neural plate is pre-patterned by global signalling gradients that induce crude AP and DV identities in neural cells [1-3]. Subsequently, groups of cells within the neuroepithelium are set aside to form local signalling centres, or 'secondary organisers', that pattern subregions of the neural tube in a spatially more restricted fashion [4-6]. The ZLI is located between the presumptive Pth and the presumptive Th and it instructs cellular fate acquisition within these two major diencephalic subdivisions by secreting the signalling factor Shh [21-23]. Our understanding as to how this important neuroepithelial organiser is established is only in its infancy.

In principle, two types of signals may regulate ZLI positioning and formation: planar signals that act within the plane of the neuroepithelium and vertical signals from underlying tissue such as the axial mesendoderm. For example, the observation that the ZLI marks the interface between the prechordal and epichordal central nervous system [43] might suggest that a vertical signal derived from the interface between the prechordal mesendoderm and the chordamesoderm induces ZLI fate in the overlying neuroectoderm. Similarly, both planar and vertical signals have been implicated in early neural plate patterning [1,2,47].

Studies in chick, mouse and zebrafish have implicated various transcription factors in determining ZLI positioning. Anteriorly, *Six3*, *Fez *and *Fezl *are expressed in the presumptive Pth while, posteriorly, the presumptive Th is marked by the expression of *Irx *genes [29,38,39]. *Fez *and *Fezl *are redundantly required for the establishment of the Pth and the ZLI [38,39]. *Six3 *and *Irx *genes are regulated by canonical Wnt signalling, raising the possibility that the AP position of the ZLI is directly determined by a specific threshold in the early Wnt/β-catenin activity gradient that polarises the AP axis of the nascent neural plate [2,3,5].

### Early specification of the ZLI

Here, we have described tissue explant experiments aimed at exploring the spatial and temporal requirements of ZLI formation. We found in both explants comprising the entire embryonic diencephalon and explants of diencephalic subregions, that the ZLI has been specified by stage HH10. It is not possible to obtain explants from younger embryos with the precision required to address questions of diencephalic regionalisation, imposing an intrinsic limitation to our experimental approach. Thus, it is possible that the AP position of the ZLI is determined even earlier in development, in line with the hypothesis that graded signals during gastrulation are directly required to induce this local signalling centre.

Cell labelling experiments *in vivo *have demonstrated that the ZLI forms from a wedge-shaped area in the early prosencephalic anlage that is characterised by the absence of expression of *Lfng *and that is enclosed by cell lineage restriction boundaries both anteriorly and posteriorly [37]. However, this model has recently been called into question [48]. *In situ *hybridisation does not allow gene expression to be mapped at the level of single cells; however, our observation that pro-ZLI explants from stage HH10 or stage HH14 embryos expressed *Shh *throughout rather than in a thin (ZLI-like) stripe after culture is consistent with our previous data showing that the entire *Lfng*-free pro-ZLI wedge gives rise to the ZLI.

### Influence of flanking tissues on ZLI formation

The mechanisms regulating ZLI morphogenesis remain unknown. It is not clear whether the transformation from a short broad structure (the *Lfng*-free wedge) into a long narrow structure (the *Shh*-expressing ZLI) is simply due to allometric growth of the pro-Pth, pro-ZLI and pro-Th regions or whether active morphogenetic processes are involved. While our pro-ZLI explants expressed *Shh *throughout and failed to undergo the elongation characteristic of ZLI morphogenesis *in vivo*, an elongated ZLI was obtained in explants comprising the entire diencephalic anlage. Neither pro-Pth + pro-ZLI nor pro-ZLI + pro-Th explants resulted in the formation of an elongated ZLI. This observation suggests that the integrity of the entire region is required to allow for proper ZLI morphogenesis. It is tempting to speculate that the lineage-restricted boundaries flanking the pro-ZLI anteriorly and posteriorly are both required as 'girders' that impose geometric restrictions on the pro-ZLI region during its morphogenesis, thereby forcing it to narrow and elongate.

*Shh *expression in the ZLI starts ventrally just next to the basal plate and progresses dorsally between stages HH15 and HH18. Based on explant experiments similar to ours it has been suggested that Shh signalling from the ventral diencephalon is required to induce *Shh *expression in the ZLI and that a cell-to-cell relay mechanism underlies the ventral-to-dorsal progression of this process [41]. Our stage HH10 explants did not include basal diencephalon, yet they recapitulated ZLI formation faithfully *in vitro*, indicating that the ZLI can form independently from ventral tissues, at least after this developmental stage. At this point, we cannot rule out a requirement for basal plate-derived signals in ZLI induction at earlier stages, as there are no experimental means to completely and reliably ablate the (prospective) basal plate at early stages in the chick embryo like in mouse or zebrafish embryos, which are amenable to classical genetic approaches. However, our laboratory has recently examined ZLI formation in the zebrafish embryo and that study supports our present findings in chick [23]. Specifically, the observation that the ZLI forms in *one-eyed pinhead *mutants, which completely lack ventral neural tissues and all ventral expression of *Shh*, calls a requirement for basal plate-derived signals into question [23].

Dorsal diencephalic tissue has been described to oppose ZLI formation [41]. We could confirm this antagonistic interaction using our explant system and found that the RA-producing enzyme CYP1B1 is expressed dorsally, in the epithalamus, during ZLI formation. Ectopic expression of *CYP1B1 *results in a downregulation of *Shh *expression in the ZLI, suggesting that RA is a good candidate signal to mediate the ZLI-inhibitory function of dorsal diencephalic tissue. This does not rule out that other dorsal signals may also contribute to this dorsal inhibition. Various factors of the Wnt family are expressed in the dorsal diencephalon and Wnts have been shown to attenuate the response of neural tissue to Shh signalling [49]. However, in preliminary electroporation experiments using activators of the Wnt pathway we never observed downregulation of *Shh *in the ZLI (data not shown). Thus, Wnts are unlikely to mediate the inhibitory function of dorsal diencephalic tissue. Bone morphogenetic proteins (BMPs) are dorsalising factors in the spinal cord [7] and *Bmp5 *is expressed in a thin stripe along the dorsal forebrain during ZLI formation [50]. Candidacy of BMP5 as a ZLI-inhibitory signal remains to be tested experimentally.

### Planar interactions resulting in ZLI formation

Using explant co-cultures and quail-chick chimeras we found that interaction between any prechordal and any epichordal neuroepithelium anterior to the MHB resulted in ZLI formation, confirming and extending the results by Vieira *et al*. [22]. In contrast to the Vieira *et al*. study, we frequently observed ectopic induction of *Shh *expression around grafts that was discontinuous with the endogenous ventral expression domain of this gene. This is consistent with our and others' findings that ventral signals are dispensable for ZLI formation. In our explant co-cultures, we observed *Shh *induction in both prechordal and epichordal tissues. Similarly, Vieira *et al*. found graft-autonomous and non-autonomous induction of *Shh *while, in our grafting experiments, *Shh *appears to be induced mostly outside of the graft in epichordal host tissue (Figure 5a–d). Different size and/or location of the grafts may account for this minor discrepancy. Taken together, these observations indicate that both prechordal and rostral epichordal (posterior diencephalon, midbrain) neural tissue are competent for ZLI induction.

The induction of ZLI formation by an interaction between anterior and posterior neuroectoderm strongly favours a planar model for ZLI induction and is highly reminiscent of the formation of a MHB following recombination of midbrain and hindbrain tissue [51,52]. It appears that the formation of local organisers along the AP axis of the neural tube is a fairly robust process such that – even after physical ablation – these structures will easily regenerate.

## Conclusion

**Figure 9:**
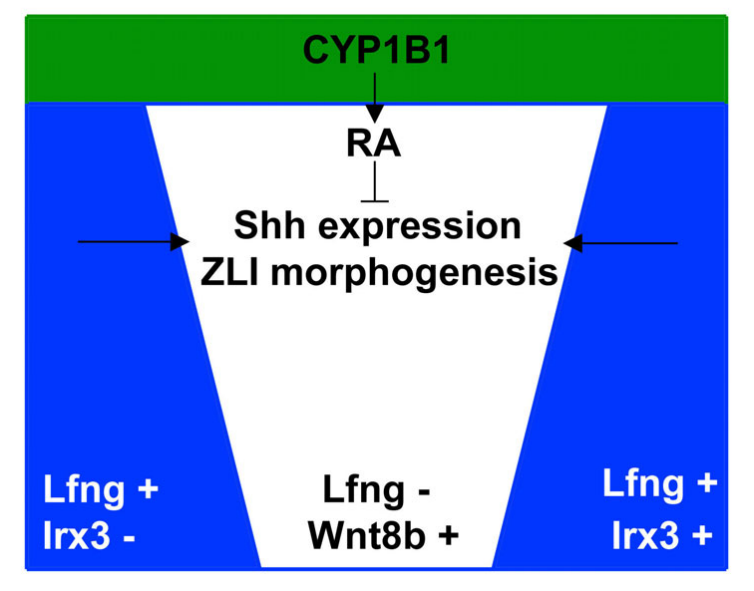
Schematic model for ZLI establishment. Morphogenesis of the *Lfng*-negative wedge and *Shh *expression in the definitive ZLI are positively regulated by planar signals from the adjacent *Lfng*-positive areas (pro-Pth, *Irx3*-negative; pro-Th, *Irx3*-positive). *CYP1B1 *is expressed in the dorsal diencephalon and generates RA that inhibits *Shh *expression in the ZLI.

We found that the ZLI becomes specified before stage HH10, that the entire forebrain area is competent to form a ZLI and that any prechordal-epichordal interaction in this area will lead to ZLI formation, consistent with a model where early planar signalling is sufficient for the establishment of this local signalling centre. ZLI formation occurs independently from ventral tissues after stage HH10 and is antagonised by signals from the dorsal diencephalon, specifically by RA that is produced by a patch of CYP1B1 activity in the epithalamus. The morphogenesis of the ZLI depends on the integrity of the diencephalic anlage, suggesting that structural features such as the boundaries flanking the pro-ZLI anteriorly and posteriorly are important to direct differential growth and/or morphogenetic movements such that an elongated, narrow stripe of *Shh*-expressing cells can form. Our results provide a basis for the ongoing identification of molecular cues underlying the process of ZLI formation by narrowing its spatiotemporal requirements. A schematic model summarising our findings is shown in Figure 9.

## Materials and methods

### Chick and quail embryos

Chick and quail eggs were obtained from Stewart Co. Ltd (Louth, UK) and Potter's farm (Huntingdon, UK), respectively, and incubated in a humidified chamber at 38°C until they reached the required stage. Staging was performed according to the tables of Hamburger and Hamilton (HH) [53]. For further manipulation, the surface of incubated eggs was disinfected using 70% ethanol.

### *In vitro *culture of neuroepithelial explants

Brain explants were dissected in sterile Tyrode's buffer using sharpened tungsten needles and were transferred to culture medium. We obtained optimal results using suspension culture in Neurobasal medium containing 2 mM Glutamax-I, 2% B27 supplement and penicillin/streptomycin (1:100; all from Invitrogen, Paisley, Scotland, UK). For co-culture experiments, explants were labelled prior to culture for 45 minutes with 0.5 nM of either red or green CellTracker reagents (Molecular Probes, Paisley, Scotland, UK) and were embedded in a collagen gel matrix as described previously [54]. Explants were cultured in a tissue culture incubator for up to three days at 37°C, 100% humidity, 5% CO_2_.

### Heterotopic quail-chick transplantation

Quail neural explants were dissected in Tyrode's buffer as described above. An incubated chick egg was windowed and the embryo was highlighted by sub-blastodermal injection of India ink (Pelikan, 1:5 in Tyrode's buffer). Extra-embryonic membranes were removed from the area of transplantation and a piece of tissue the same size and shape as the graft was excised from the neural tube. Subsequently, the graft was pasted into the resulting gap, the egg was re-sealed with sticky tape and incubated in a humidified chamber for the appropriate time.

### *In situ *hybridisation

*In situ *hybridisation was performed as described elsewhere [55]. *In situ *hybridisations of tissue explants were performed according to the same protocol, but in 35 mm culture dishes (Cellstar, Greiner, Stonehouse, Scotland, UK) with reduced solution volumes.

### Immunocytochemical detection of grafted quail cells

After *in situ *hybridisation of chimaeric quail-chick embryos, the specimens were re-fixed in 4% paraformaldehyde for 2 h followed by two washes with phosphate-buffered saline (PBS) + 0.1% Tween20 and one wash with PBS. Embryos were then blocked for 1 h in PBS + 10% newborn calf serum (NCS) + 1% Triton X-100 and incubated with the quail-specific antibody (QCPN; 1:10) overnight at 4°C. On the following day, the specimens were washed six times for 2 h with PBS + 1% NCS + 1% Triton X-100 and incubated overnight with the secondary antibody (Alexa green, 1:200; Molecular Probes). Six further washes were performed on the third day before the specimens were mounted for examination under the UV microscope.

### *In ovo *electroporation

Stage HH16 embryos were electroporated as described [54] with an expression plasmid for *CYP1B1 *that also drives expression of a green fluorescent protein via an internal ribosome entry site [44].

## Competing interests

The author(s) declare that they have no competing interests.

## Authors' contributions

MFG participated in planning the experiments and carried out the explant (co-) culture assays and most of the *in situ *hybridisations. DC isolated chick *CYP1B1*, performed the *in situ *hybridisation of this gene and subcloned it into an expression vector. AL provided the funding for this research, and both initiated and coordinated the study. CK participated in the design and coordination of the study, performed the *in ovo *electroporation experiments, some of the *in situ *hybridisations and explant assays and drafted the manuscript.
